# Alcohol based surgical prep solution and the risk of fire in the operating room: a case report

**DOI:** 10.1186/1754-9493-2-10

**Published:** 2008-04-26

**Authors:** Sumit Batra, Rajiv Gupta

**Affiliations:** 1 Department of Orthopaedic Surgery, Central Institute of Orthopaedics, Vardhaman Mahavir Medical College & Safdarjung Hospital, New Delhi – 110029, India

## Abstract

A few cases of fire in the operating room are reported in the literature. The factors that may initiate these fires are many and include alcohol based surgical prep solutions, electrosurgical equipment, flammable drapes etc. We are reporting a case of fire in the operating room while operating on a patient with burst fracture C6 vertebra with quadriplegia. The cause of the fire was due to incomplete drying of the covering drapes with an alcohol based surgical prep solution. This paper discusses potential preventive measures to minimize the incidence of fire in the operating room.

## Case presentation

A 25 years old male patient with burst fracture of C6 vertebra with quadriplegia was taken to the operating room for anterior decompression with fusion. He was positioned supine on the operating table. The neck, shoulder region and the upper part of chest up-to the level of nipples was shaved. General anesthesia was induced and the patient was intubated for intra-operative ventilation using closed circuit. The neck, shoulder and chest region was painted with Cutasept^R ^(contains Benzalkonium chloride and Isoproponol 63%). The surgical site was draped with cotton sheets. A longitudinal incision was given along the anterior border of sternomastoid and the subcutaneous tissue was exposed. Electrocautery was then brought into the field for deeper dissection. Flames were noticed around the surgical site immediately after the activation of the electrocautery. The flames were spread over the entire surgical field corresponding to the area prepared with Cutasept^R^. The drapes were immediately removed and the electrocautery was switched off. The fire was rapidly extinguished within seconds. The patient suffered minor burns in the neck and chest region (first degree) and recovery was uneventful.

## Discussion

Operating room fires are uncommon. Studies by Emergency Care Research Institute have shown that approximately 100 operating room fires occur every year with 10–20 of these events deemed "serious" and two directly resulting in death. Nearly 70% of these fires are related to the use of electro surgical equipment. Furthermore, in 72% of cases, an oxygen-enriched atmosphere has been shown to have contributed to the fire. It has also been noticed that there is a significant risk of fire when alcohol based surgical prep solutions are used for skin preparation [1-3]. The fact that alcohol based antiseptic solutions can provide fuel for surgical fire has been demonstrated both by reports of surgical fires and laboratory studies [4,5].

The fire triangle is a useful construct that describes the three elements necessary for initiation of a fire i.e., heat, fuel and an oxidizer. In the case of operating room fires, an electrosurgical unit most often provides heat to ignite the flammable substance, although lasers as well as fibreoptic light sources are potent heat sources as well. Fuels are abundant in the operative field that include prep solutions, drapes, sponges, endotracheal tubes, petroleum based ointments, tinctures, as well as many others. In the presence of a high oxygen environment, all of these substances can burst into flames and burn intensely [6]. Experimental studies have shown that hot wire cautery or diathermy generates enough heat to ignite all alcohol based antiseptics even if these contain as little as 20% alcohol. The ignition temperatures for these fluids are within the range of 800–900°C. These temperatures are easily reached with the use of typical electrocautery units [5].

It has been suggested that fires may be initiated by ignition of the vapor generated from the antiseptic solution. The heat of the skin ensures that this is in high concentration near the operating site. The potential for fire is augmented when the alcohol based skin antiseptic is applied in ways that allow the solution to run off and wick into the patient's hair, pool on the skin, or wick into the surrounding linen. Pleats or air pockets in the drape materials allow the flammable vapors to accumulate near the surgical site. Attention to these details demands prolongation of the required drying time. If the patient is draped before the solution is completely dry, alcohol vapors can be trapped and channeled to the surgical site, where a heat source can ignite the vapors [7].

It has been recommended that to prevent fire, removal of the fuel is the most reasonable and efficacious approach. This includes shaving the skin especially in hirsute patients to prevent pooling of solution in the hair. In all cases, significant attention must be paid to effectively draping the patient to prevent collection of flammable vapors beneath the drapes [6]. Further, the use of alcohol based antiseptics calls for strict adherence to the proper use of these substances, including observation of required drying time. This may take a few minutes or more until the field is completely dry [3]. It has also been recommended that whenever electrocautery or diathermy is to be used on or near the body surface, one of many aqueous based antiseptics be used, or if an alcohol based antiseptic is used this should be cleaned off with a dry swab before the diathermy is used [5].

It has also been recommended that a fuel-oxidizer combination is to be avoided in or near the surgical field. This is important especially in head-neck procedures in which the patient's trachea is not intubated (mask anesthesia). In such cases oxygen delivery should be kept to the minimum required to keep the oxygen saturation of blood within an acceptable range. Also the face should not be covered with drapes to allow for dissemination of exhaled oxygen to prevent pooling of oxygen under the drapes [4,6].

**Figure 1:**
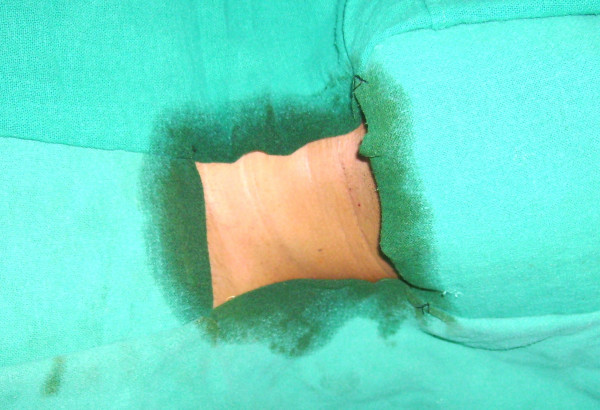
Wet margins of covering drapes even after complete drying of skin can lead to continuous formation of vapor near the surgical site.

A number of alcohol based surgical prep solutions are commercially available and are being used on regular basis e.g., Duraprep^R ^(contains 0.7% available iodine and 74% isopropyl alcohol), Chlorolprep ^R ^(contains 2% chlorhexidine gluconate and 70% isopropyl alcohol), Scrubcare brand Prevail-Fx^R ^(contains 0.83% available iodine and 72.5% isopropyl alcohol). In our case we had used Cutasept^R ^that contains isoproponol (63%). We observed that the margins of the drapes remained wet even after the operating field was dried with a swab and that could have lead to a collection of vapors near the skin (Figure 1). As soon as the cautery was activated the vapors immediately catched fire. Hence, it is recommended that draping should be done after complete drying of the antiseptic solution. Also, covering the surgical site and the drape margins with a clear plastic adhesive drape would prevent vapor leakage and collection at the surgical site.

The case was presented and discussed in the monthly inter-departmental clinical meeting in the hospital and the experience was shared with other surgical specialities. As a precaution, whenever Cutasept^R ^is used for skin preparation, we strictly wait for three minutes for the solution to dry and the skin is wiped with a cotton swab before draping the surgical site. A plastic adhesive drape is also being used in every case for covering the surgical site before the surgery. The use of Iodine which is less flammable than alcohol as an alternative surgical prep solution is being increased to reduce the risk of such a mishap in the future.

It is difficult to eliminate all the risk factors of operating room fires, but we can minimize the risk by educating the surgeons, anesthetists and other operating room personnel on this subject and taking adequate precautions.

## Conclusion

Alcohol based surgical prep solutions can ignite with the use of electrocautery and lead to fire in the operating room. For prevention of such a catastrophic event it is recommended that the alcohol based solution should be dried properly before draping the surgical site. Covering the skin with a clear plastic adhesive drape also reduces the collection of alcohol vapor at the site, thus reducing the chances of fire.

## Competing interests

The authors declare that they have no competing interests.

## Authors' contributions

SB was the operating surgeon and has written the case report. RG has collected the references and has helped in writing the case report. All authors have read and approved the final manuscript.

## Consent

Written consent was obtained from the patient for publication of study.
